# Phylogenetic Relationships of Plant Bugs Based on Mitochondrial Genomes (Heteroptera: Miridae)

**DOI:** 10.1002/ece3.73035

**Published:** 2026-02-04

**Authors:** Jia‐Dong Yin, Bo‐Lun Cai, Wen‐Jun Bu, Qiang Xie

**Affiliations:** ^1^ School of Life Sciences, State Key Laboratory of Biocontrol Sun Yat‐sen University Guangzhou Guangdong China; ^2^ School of Ecology, State Key Laboratory of Biocontrol Sun Yat‐Sen University Shenzhen Guangdong China; ^3^ College of Life Sciences Nankai University Tianjin China

**Keywords:** Bryocorinae, high‐throughput sequencing, Miridae, mitochondrial genome, molecular phylogeny

## Abstract

Miridae is the most species‐rich family of true bugs and plays an important role in both natural and agricultural ecosystems. However, contemporary controversies surrounding their phylogenetic relationships and subfamily classification still lack consensus. This study employs molecular systematics to resolve Miridae phylogeny, utilizing mitochondrial genomes from 42 species spanning 39 genera across six of the seven currently recognized subfamilies. Four outgroup species from Tingidae (2 species) and Thaumastocoridae (2 species) were also included in the analyses. Our results demonstrate that: (1) Bryocorinae is paraphyletic as the stem groups of Miridae; and (2) the clade ((Deraeocorinae + Mirinae) + (Orthotylinae + Phylinae)) is consistently and strongly supported as a monophyletic group across all datasets and analytical methods. We report newly sequenced mitochondrial genomes based on high‐throughput sequencing platforms for four Miridae genera and species: *Chlamydatus* sp. (Phylinae), *Deraeocoris punctulatus* (Deraeocorinae), *Scirtetellus* sp. (Orthotylinae), and *Prodromus clypeatus* (Bryocorinae). These findings provide a progressive phylogenetic framework with new significance for the future phylogenetic improvement and taxonomic revision of Miridae.

## Graphical abstract

This study for the first time employs mitochondrial genomes to resolve Miridae phylogeny, with 42 species spanning 39 genera across six of the seven currently recognized subfamilies. Bryocorinae is paraphyletic as the stem groups of Miridae. These findings provide a progressive phylogenetic framework with new significance for the future phylogenetic improvement and taxonomic revision of Miridae, which is the most species‐rich family of true bugs and plays an important role in both natural and agricultural ecosystems.

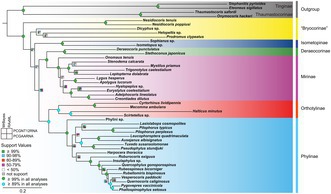

## 1 Introduction

The Miridae, commonly referred to as plant bugs, are classified within the order Hemiptera and suborder Heteroptera. As the largest and most diverse group of true bugs, they comprise more than 11,700 described species across seven subfamilies, 44 tribes, and more than 1450 genera (Schuh 1995; Schuh and Weirauch 2020). This hyperdiverse group exhibits remarkable ecological plasticity, occupying a wide range of niches as phytophages, predators, zoophytophages, and fungivores (Wheeler 2001; Cassis and Schuh 2012). Given their significant roles in both natural ecosystems and agroecosystems, where they function either as major pests or as biological control agents, understanding their phylogenetic relationships is fundamental to unraveling the evolutionary mechanisms underlying their extraordinary diversity (Aviron et al. 2016; Oh et al. 2023; Chen et al. 2024).

**FIGURE 1:**
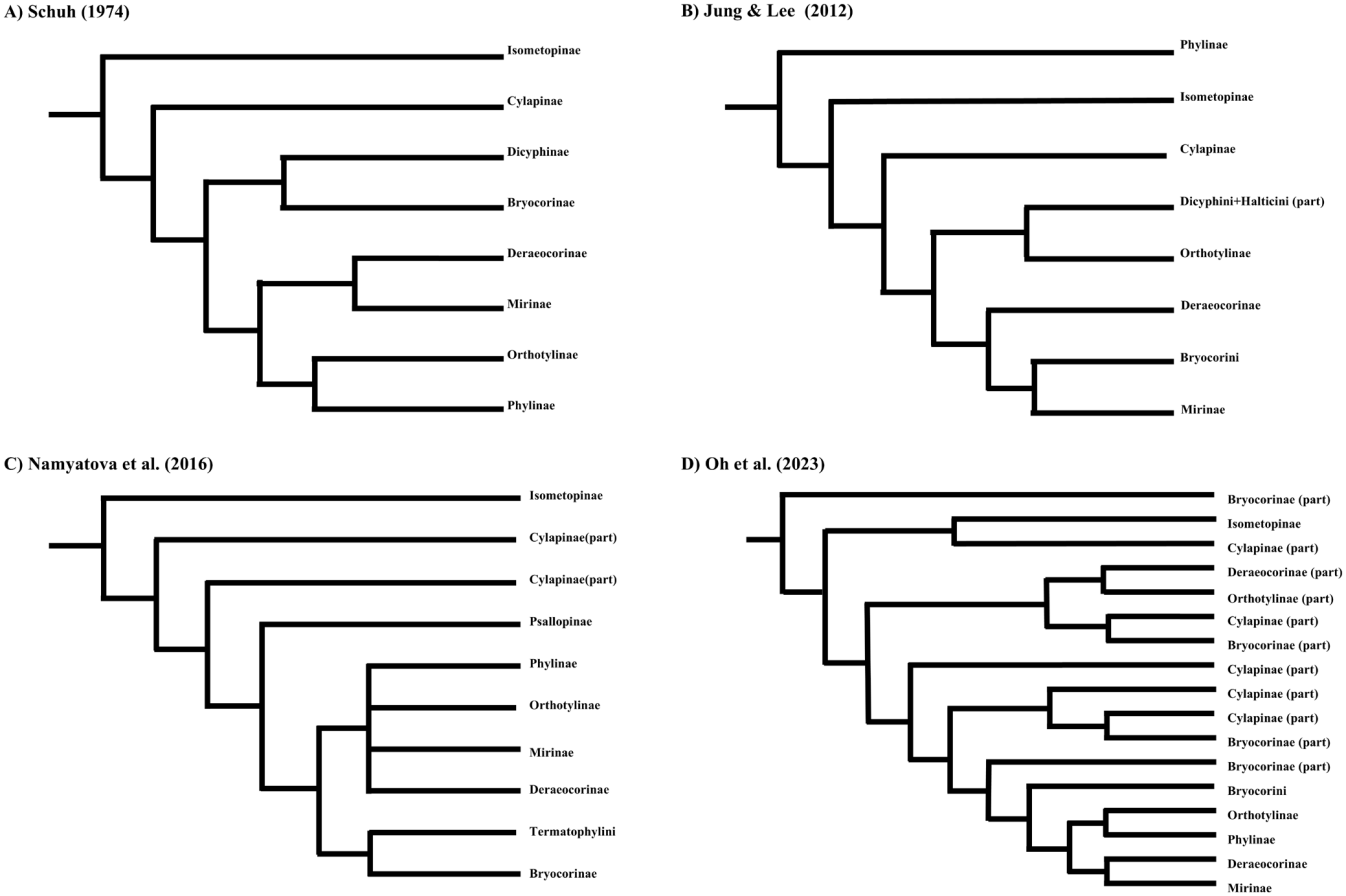
History of changes on higher‐level phylogenetic hypotheses of Miridae. (A) Schuh (1974); (B) Jung and Lee (2012); (C) Namyatova et al. (2016); (D) Oh et al. (2023).

The phylogenetic reconstruction of Miridae has changed significantly over time. In the last century, it was primarily based on morphological characters. Foundational studies relied heavily on features such as pretarsal structures, trichobothria patterns, and genitalia. A series of sequential studies by Schuh in the 1970s significantly advanced the understanding of Miridae systematics. In 1974, Schuh established the phylogenetic relationships among mirid subfamilies by using 13 key morphological characteristics (Figure 1). Subsequently, Schuh (1976) expanded on this foundation by updating and confirming the classification of Miridae, recognizing seven subfamilies, including the establishment of the new subfamily Psallopinae, and proposing a cladistic framework based on pretarsal structures. At the same time, it was also determined that there is a sister group relationship between Miridae and Deraeocorinae.

A major turning point occurred with the introduction of molecular techniques at the beginning of the 21st century. Schuh et al. (2009) conducted comprehensive total‐evidence analyses that combined morphological and molecular data (16S, 18S, 28S, *COI*). Their work recovered the superfamily Miroidea (Miridae + Tingidae + Thaumastocoridae) and revealed the non‐monophyly of Bryocorinae within the molecular partitions. Jung and Lee (2012) expanded the taxon sampling and confirmed this result, while also controversially suggesting Phylinae as the earliest‐diverging subfamily (Figure 1). Following this, research focus shifted to resolving internal relationships within specific subfamilies. For instance, Menard et al. (2014) redefined tribal classifications within Phylinae using combined datasets, synonymizing Auricillocorini with Hallodapini and establishing new tribes like Decomini. Concurrently, Namyatova et al. (2016) utilized morphological evidence to propose an updated tribal classification for Bryocorinae, elevating a formal subtribe to tribe status and erecting the new tribe Felisacini. Further studies on Deraeocorinae and Mirinae continued to reveal non‐monophyletic patterns within traditionally recognized tribes such as Deraeocorini and Mirini (Kim and Jung 2019; Kim et al. 2022). Most recently, Oh et al. (2023) incorporated novel nuclear markers (histones H2A, H3A) and estimated divergence times, placing the origin of Miridae in the Late Jurassic (~163.4 Ma) and linking major subfamily radiations to the diversification of Cretaceous angiosperms. Phylogenetically, their multi‐locus analysis did not support the monophyly of Bryocorinae; instead, Bryocorinae, Cylapinae and Deraeocorinae were recovered as polyphyletic, comprising two to five distinct clades (Figure 1). In contrast, the analysis strongly supported the sister‐group relationship between Orthotylinae and Phylinae, a topology consistent with earlier morphological hypotheses.

Despite substantial progress, significant challenges and unresolved issues remain in the phylogenetics of Miridae. Methodologically, although commonly used genetic markers (e.g., *COI*, 16S, 18S, 28S) have provided valuable insights, there is a recognized need to explore novel genetic markers and potentially phylogenomic‐scale datasets to increase resolving power, especially for deep nodes in the phylogeny (Knyshov et al. 2019).

In parallel with morphological and multi‐locus nuclear data, mitochondrial genomes have emerged as valuable tools for phylogenetic reconstruction within Miridae, offering insights into genomic architecture and evolutionary patterns. Early mitogenomic studies in mirids were limited, but recent advances have provided complete sequences for several key species, revealing conserved features and potential synapomorphies. The complete mitogenome of *Creontiades dilutes* (Stål, 1859) (green mirid) was reported by Hereward (2016). It is 15,864 bp in length and exhibits the typical insect mitochondrial gene arrangement, containing 13 protein‐coding genes (PCGs), 22 tRNAs, two rRNAs, and a control region. This work contributed to the growing body of mitogenomic data for Miridae, highlighting the utility of these genomes for phylogenetic inference and suggesting a close relationship between *Creontiades* Distant, 1883, and *Adelphocoris* Reuter, 1896 within Mirinae.

Subsequent work by Tan et al. (2018) on *Lygus pratensis* (Linnaeus, 1758) described a 16,591 bp mitogenome in which all PCGs used standard start codons (ATN) and most possessed complete stop codons (TAA), though some exhibited incomplete stop codons (e.g., “T–” for *COI*). The tRNAs displayed classic cloverleaf secondary structures, and the control region (2017 bp) contained tandem repeats. Phylogenetic analysis based on mitochondrial genome data supported the monophyly of Miridae and showed *L. pratensis* clustering with other *Lygus* Hahn, 1833 species, closely related to *Apolygus* China, 1941 and *Adelphocoris*. More recently, Chen et al. (2024) sequenced the complete mitogenome of *Stethoconus japonicus* Schumacher, 1917 (Deraeocorinae), which was 16,274 bp with an AT content of 73.49%. The study emphasized evolutionary traits such as AT bias, conserved gene order, and repetitive elements in the control region, while phylogenetic analysis indicated Deraeocorinae is closely related to Mirinae rather than Bryocorinae.

These studies collectively demonstrate that mirid mitogenomes are highly conserved in structure and gene content, typically showing strong AT bias and characteristic features like tandem repeats in the control region. They have proven valuable for resolving phylogenetic relationships at various taxonomic levels, from species to subfamilies. Additionally, methods like PCR‐generated bait capture (Knyshov et al. 2019) have facilitated the sequencing of mitogenomes from museum specimens, expanding the potential for historical and comparative genomics. However, the phylogenetic relationships within Miridae based on mitochondrial genomes have not yet been thoroughly investigated.

To address this gap and to help clarify the phylogenetic relationships within Miridae, this study sequenced the complete mitochondrial genomes (mitogenomes) of four mirid species, each representing one of the subfamilies Bryocorinae, Orthotylinae, Deraeocorinae, and Phylinae, and systematically characterized their genomic features. For the first time, phylogenetic analyses were conducted using both maximum likelihood (ML) and Bayesian inference (BI) methods based on this mitogenomic data. Our results indicate that the subfamilies Deraeocorinae, Mirinae, Orthotylinae, and Phylinae are each monophyletic and together form a well‐supported monophyletic group. Furthermore, with Bryocorinae occupying the basal position within the family, it was recovered as paraphyletic.

## 2 Materials and Methods

### 2.1 Taxon Sampling and DNA Extraction

The taxonomic sampling in this study included a total of 46 species. Specifically, the ingroup consisted of 42 species distributed across six subfamilies: 11 from Mirinae, 5 from Bryocorinae, 4 from Orthotylinae, 2 from Deraeocorinae, 2 from Isometopinae, and 18 from Phylinae (Table 1). The outgroup comprised four species representing the families Thaumastocoridae (*n* = 2) and Tingidae (*n* = 2). The ingroup covered six out of the seven subfamilies of Miridae. The mitochondrial genomes of four Miridae genera and species, *Chlamydatus* sp. (Phylinae), *Deraeocoris punctulatus* (Rambur, 1839) (Deraeocorinae), *Scirtetellus* sp. (Orthotylinae), and *Prodromus clypeatus* Distant, 1904 (Bryocorinae), were newly sequenced in this study. Specimens were collected from four distinct localities of China: (1) Ngari Prefecture, Xizang (31°17′ N, 84°58′ E), (2) Burqin County, Xinjiang (48°0′ N, 86°49′ E), (3) Tashkurgan Tajik Autonomous County, Xinjiang (36°51′ N, 75°31′ E), (4) Xishuangbanna Tropical Botanical Garden, Yunnan (21°54′ N, 101°17′ E). All samples were preserved in 100% ethanol at −20°C until DNA extraction. Genomic DNA was extracted from the head and thorax with the TIANamp Genomic DNA Kit (Tiangen Biotech Co. LTD., Beijing, China) following the manufacturer's instructions.

**TABLE 1:** Taxon sampling in this study.

Subfamily	Species	Accession number of mitogenome
Mirinae	*Lygus hesperus*	NC_024641
Mirinae	*Creontiades dilutus*	NC_030257
Mirinae	*Adelphocoris lineolatus*	NC_027143
Mirinae	*Apolygus lucorum*	NC_023083
Mirinae	*Trigonotylus caelestialium*	KJ170899
Mirinae	*Stenodema calcarata*	OY986066
Mirinae	*Leptopterna dolabrata*	OX940991
Mirinae	*Onomaus tenuis*	MW619699
Mirinae	*Hyalopeplus* sp.	MW619698
Mirinae	*Mystilus priamus*	MW619697
Mirinae	*Eurystylus coelestialium*	MK251132
Bryocorinae	*Nesidiocoris tenuis*	NC_022677
Bryocorinae	*Nesidiocoris poppiusi*	OR099830
Bryocorinae	*Dicyphus* sp.	MW619696
Bryocorinae	*Helopeltis* sp.	MW619695
Bryocorinae	Prodromus clypeatus*	PX853040
Orthotylinae	*Halticus minutus*	NC_061220
Orthotylinae	*Cyrtorhinus lividipennis*	NC_064994
Orthotylinae	*Mecomma ambulans*	MW619692
Orthotylinae	*Scirtetellus* sp.*	PX853039
Deraeocorinae	*Stethoconus japonicus*	NC_069555
Deraeocorinae	*Deraeocoris punctulatus* *	PX853038
Isometopinae	*Isometopus* sp.	MW619700
Isometopinae	*Sophianus* sp.	MW619713, MW619712, MW619711
Phylinae	*Harpocera thoracica*	OZ187107
Phylinae	*Pilophorus perplexus*	OY037020
Phylinae	*Pilophorus typicus*	MW619694
Phylinae	*Lasiolabops cosmopolites*	MW619693
Phylinae	*Pseudophylus stundjuki*	MK393986
Phylinae	*Leucophoroptera quadrimaculata*	MK394001
Phylinae	*Tuxedo susansolomonae*	MK393999
Phylinae	*Ausejanus albisignatus*	MK393985
Phylinae	*Rubellomiris bispinosus*	MK393976
Phylinae	*Insulaphylus* sp.	MK393948
Phylinae	*Phallospinophylus setosus*	MK393974
Phylinae	*Pygovepres vaccinicola*	MK393961
Phylinae	*Vesperocoris paddocki*	MK393949
Phylinae	*Quernocoris caliginosus*	MK393947
Phylinae	*Rubeospineus bicorniger*	MK393946
Phylinae	*Quercophylus gonoporospinus*	MK393944
Phylinae	*Roburocoris exiguus*	MK393943
Phylinae	*Chlamydatus* sp.*	PX853037
Thaumastocorinae	*Thaumastocoris safordi*	MW619691
Thaumastocorinae	*Onymocoris hackeri*	MW619690
Tinginae	*Eteoneus sigillatus*	NC_086666
Tinginae	*Stephanitis pyrioides*	NC_085677

*Note:* The asterisk indicates newly sequenced mitogenomes in this study.

### 2.2 Genome Sequencing, Assembly, Annotation and Analysis

Genomic DNA was sequenced using high‐throughput sequencing (HTS) technology. Four independent libraries were constructed with an average insert size of 250 base pairs (bp) and sequenced on the BGISEQ‐T7 platform (Biomarker Technologies, Beijing, China) with 150 bp paired‐end (PE) reads. Raw reads were quality‐filtered to remove adaptor contamination, poly‐N sequences (containing > 5 Ns), and PE reads with more than 10 bases of low‐quality scores (< 20). After data filtration, we obtained 25.68–29.1 Mb clean reads per species. To minimize errors introduced during the assembly process, two independent methods were used for de novo assembly of complete mitogenomes for each species. The first method employed SOAPDENOVO2 for de novo assembly (Luo et al. 2012), followed by a local database comparison to identify mitogenome sequences using the BLAST+ program (Camacho et al. 2009). The other mitogenomes used to construct the local database were downloaded from the NCBI database (https://www.ncbi.nlm.nih.gov/). The second method utilized MITOBIM to directly assemble and bait mitogenomes, using closely related species as references (Hahn et al. 2013).

The MITOS2 web server was used for mitogenome annotation with the invertebrate genetic code and RefSeq63 Metazoa reference data (Bernt et al. 2013). Protein‐coding gene (PCG) boundaries were re‐examined using the Open Reading Frame Finder (ORF Finder; https://www.ncbi.nlm.nih.gov/orffinder). Additionally, PCG boundaries were validated by comparison with homologous genes from published mitogenomes of Heteroptera species. Boundaries of the 12S and 16S ribosomal RNA (rRNA) genes were identified based on the flanking *tRNA*‐*Leu* (L1) and *tRNA*‐*Val* (V) genes, and further confirmed by comparison with corresponding regions in known Heteroptera mitogenomes.

Base composition and relative synonymous codon usage (RSCU) values were calculated using MEGA 11 (Tamura et al. 2021). Base composition skews were analyzed using the AT‐skew [(A‐T)/(A + T)] and GC‐skew [(G‐C)/(G + C)] formulas (Perna and Kocher 1995). To assess the evolutionary rates of mitochondrial PCGs, the non‐synonymous substitution rate (Ka), synonymous substitution rate (Ks), and the Ka/Ks ratio were calculated for each PCG using DnaSP 6 (Rozas et al. 2017). Compositional heterogeneity among sequences was evaluated with ALIGROOVE (Kück et al. 2014).

### 2.3 Phylogenetic Analysis

The phylogenetic relationships within the family Miridae were reconstructed using 37 mitochondrial genes. The amino acid sequences of 13 PCGs and RNA genes were individually aligned using MAFFT with the L‐INS‐i algorithm (Katoh and Standley 2013). The corresponding nucleotide sequence alignments were performed using TranslatorX (Talavera and Castresana 2007). Subsequently, ambiguously aligned sites were trimmed using Gblocks (Abascal et al. 2010). Substitution saturation of nucleotides was assessed by DAMBE (Xia 2013), the substitution of the third codon position of the PCGs was detected as saturated (NumOTU = 32, Iss = 0.702, Iss. cAsym = 0.607; *p* < 0.01), and thus the nucleotides in the third codon positions were excluded from the matrix. Following this, the PCGs and RNA genes were concatenated into two datasets: (Abascal et al. 2010) the PCGNT12RNA dataset, which included the first and second codon positions of all 13 PCGs, 22 transfer RNAs (tRNAs) and 2 rRNAs; (Aviron et al. 2016) the PCGAARNA dataset, comprising the amino acid sequences of the 13 PCGs and the nucleotide sequences of tRNAs and rRNAs (Data S1 and S2).

For both datasets, substitution models and partitioning schemes were examined using IQ‐TREE (Nguyen et al. 2015). The best‐fit models and partitioning schemes are summarized in Table S1. Bayesian Inference (BI) and Maximum Likelihood (ML) analyses were performed using MrBayes 3.2 and RAxML 8 (Ronquist et al. 2012; Stamatakis 2014), respectively. For the BI analyses, two independent runs were executed, each running for 2,000,000 generations and sampling every 100 generations, with a burn‐in of the first 25% generations. In the ML analyses, node support values were calculated via 2000 ultra‐fast bootstrap replicates.

## 3 Results

### 3.1 Mitogenome Composition of Miridae

**FIGURE 2:**
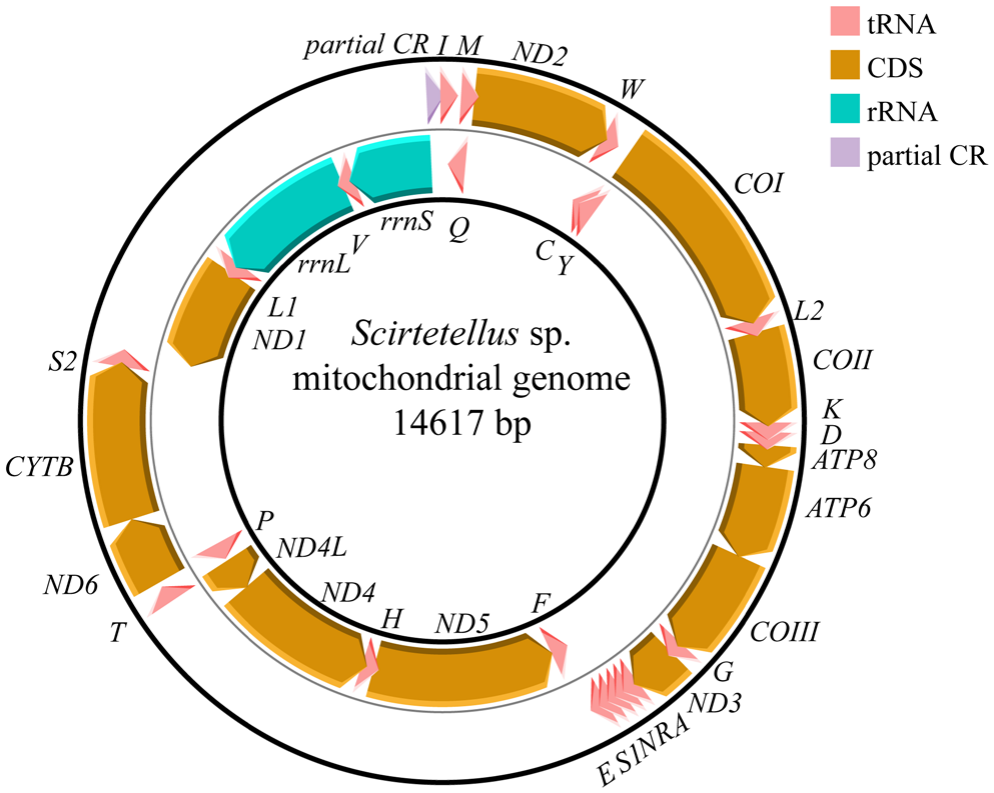
Circular diagram of the *Scirtetellus* sp. mitogenome, including control region. The transcriptional direction is denoted by arrows.

In the NCBI database, 29 Miridae species have complete mitochondrial genome sequences, with their full lengths (excluding the control region, CR) ranging from 14,267 to 15,095 bp. The mitogenomes of four newly sequenced Miridae genera and species range in size from 14,484 to 14,926 bp (Table 2), which is consistent with the available mitochondrial genome data in the NCBI database. Each genome contains the 37 typical genes, including 13 PCGs, 22 tRNAs, and 2 rRNAs. All four mitogenomes exhibit an identical gene distribution pattern across the coding strands: 23 genes are located on the majority strand, while the remaining 14 genes are found on the minority strand (Figure 2 and Figure S1). Overall, the total lengths of these mitogenomes display relatively conserved characteristics among the four newly sequenced Miridae genera and species.

**TABLE 2:** Length of Miridae mitochondrial genomes, AT‐skew and GC‐skew were measured for the 37 genes.

Species	PCGs (bp)	tRNAs (bp)	12S rRNA (bp)	16S rRNA (bp)	Total (bp)	AT‐skew	GC‐skew
*Chlamydatus* sp.	10,937	1437	784	1231	14,498	0.16	−0.19
*Deraeocoris punctulatus*	11,034	1464	796	1250	14,926	0.14	−0.16
*Scirtetellus* sp.	11,027	1472	758	1241	14,516	0.11	−0.17
*Prodromus clypeatus*	11,064	1437	753	1239	14,484	0.09	−0.16

The mitogenomes of the four Miridae species exhibit a nucleotide composition biased toward A/T, with A + T content ranging from 74.2% to 77.8% (Table S2). All mitogenomes show moderate A‐skew (0.09–0.16) and C‐skew (−0.16 to −0.19), consistent with previous findings on mitogenomes of other Miridae species. The total length of the 13 PCGs ranges from 10,937 bp in *Chlamydatus* sp. to 11,064 bp in *P. clypeatus* (Table 2). The A + T contents of the 13 PCGs vary between 73.5% (*Chlamydatus* sp.) and 76.9% ( *P. clypeatus* ). The predominant start codons are ATG and ATT, while the stop codons commonly include TAA and TAG, along with the incomplete stop codon T. The most frequently used codons are UUA (L), CCU (P), UCU (S), AUA (M), UGA (S), and UAU (Y), while those with relatively low usage frequencies include GCG (A), CCG (P), and ACG (T) (Figure S2). The A + T contents of tRNAs range from 75.9% (*Chlamydatus* sp.) to 79.5% ( *P. clypeatus* ).

The 12S and 16S rRNA genes in the four Miridae species are encoded on the J‐strand (the majority strand of the mitochondrial genomes, which serves as the primary template for transcription and replication and encodes most protein‐coding genes and structural RNAs in Hemipteran mitogenomes), with the 16S rRNA gene situated in the conserved region between *L1* and *V*, and the 12S rRNA gene located downstream of *V*. The length of the 12S rRNA gene varies from 753 bp in *P. clypeatus* to 796 bp in *D. punctulatus* , with A + T content ranging from 78.6% ( *D. punctulatus* ) to 81.7% ( *P. clypeatus* ). The 16S rRNA gene ranges from 1231 bp in *Chlamydatus* sp. to 1250 bp in *D. punctulatus* , with A + T content varying between 78% ( *D. punctulatus* ) and 80.2% ( *P. clypeatus* ). Overall, the 12S and 16S rRNA genes among the four Miridae species show little variation in size or A + T content (Table 2).

The Ka/Ks ratios were calculated to assess the evolutionary rates of the 13 PCGs in Miridae species (Figure S3). The results showed that all average Ka/Ks ratios were less than 1, ranging from 0.050 (COI) to 0.818 (ATP8), indicating that these PCGs have undergone purifying selection (Ye et al. 2021). Notably, the average Ka/Ks ratios for four genes, *COI*, *COII*, *COIII*, and *CYTB* (0.050, 0.136, 0.143, 0.101, respectively), were significantly lower than those of the remaining genes, suggesting that these four genes are generally subject to stronger selective pressure and greater evolutionary constraints (Figure S4).

**FIGURE 3:**
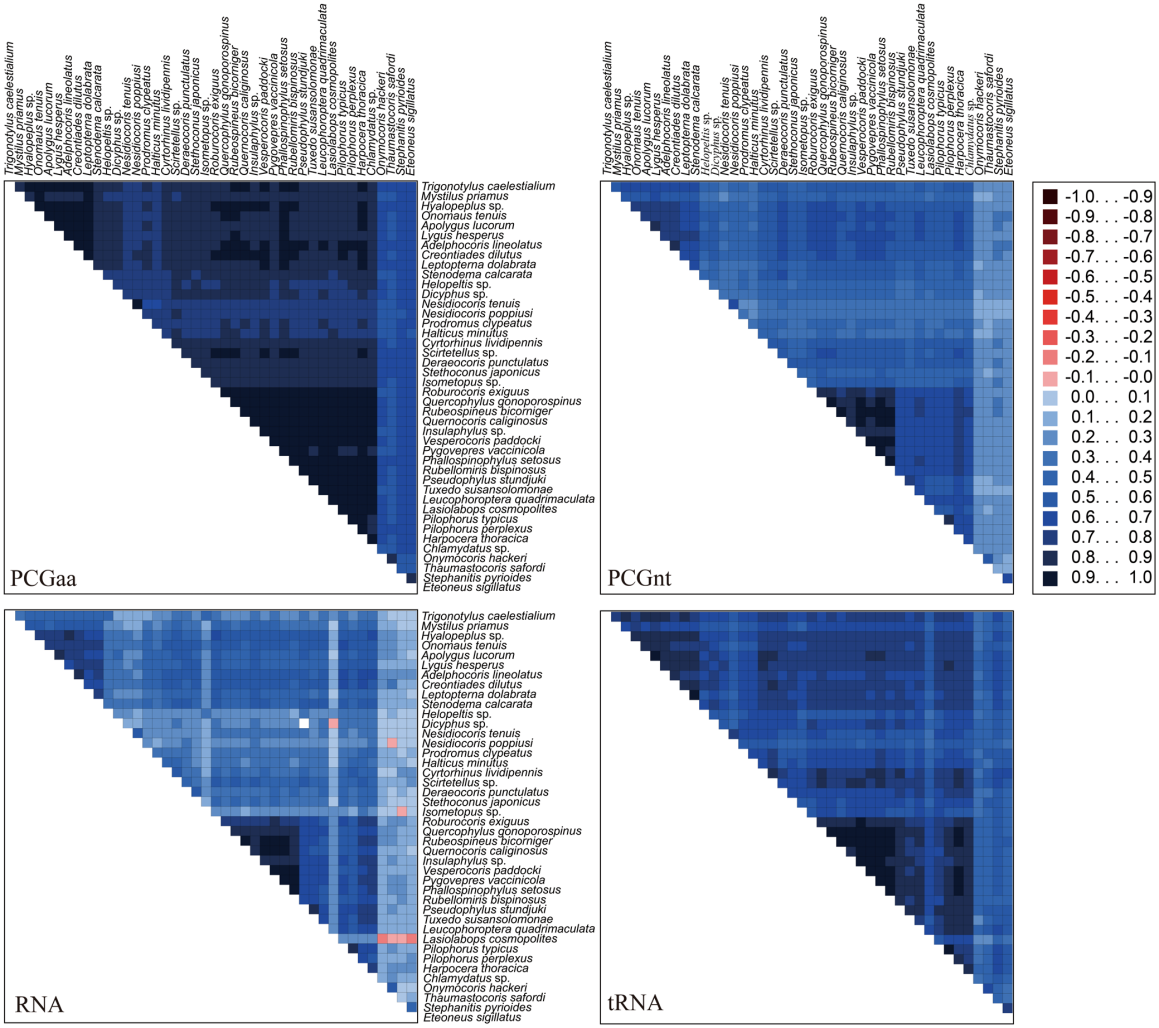
Compositional heterogeneity of mitochondrial genome sequences used in phylogenetic analyses. The average similarity scores between sequences are represented by colored squares, based on the AliGROOVE scoring system: Ranging from −1 (red) indicating significant divergence in evolutionary rate compared to the rest of the dataset, to +1 (blue) indicating matching rates with all other compared sequences.

Compositional heterogeneity in amino acid or nucleotide sequences can potentially affect the accuracy of likelihood‐based phylogenetic tree reconstruction (Lockhart et al. 1994). Since the four sequences downloaded from the NCBI database (*Sophianus* sp., *Eurystylus coelestialium* (Kirkaldy, 1902), *Mecomma ambulans* (Fallén, 1807), *Ausejanus albisignatus* (Knight, 1938)) contained substantial amounts of missing data, they were removed prior to AliGROOVE analyses. The AliGROOVE results showed that both nucleotide and amino acid sequences of the PCGs exhibited low levels of compositional heterogeneity (Figure 3). Therefore, our phylogenetic analyses were unlikely to have been significantly affected by sequence heterogeneity.

### 3.2 The Phylogeny of Miridae

**FIGURE 4:**
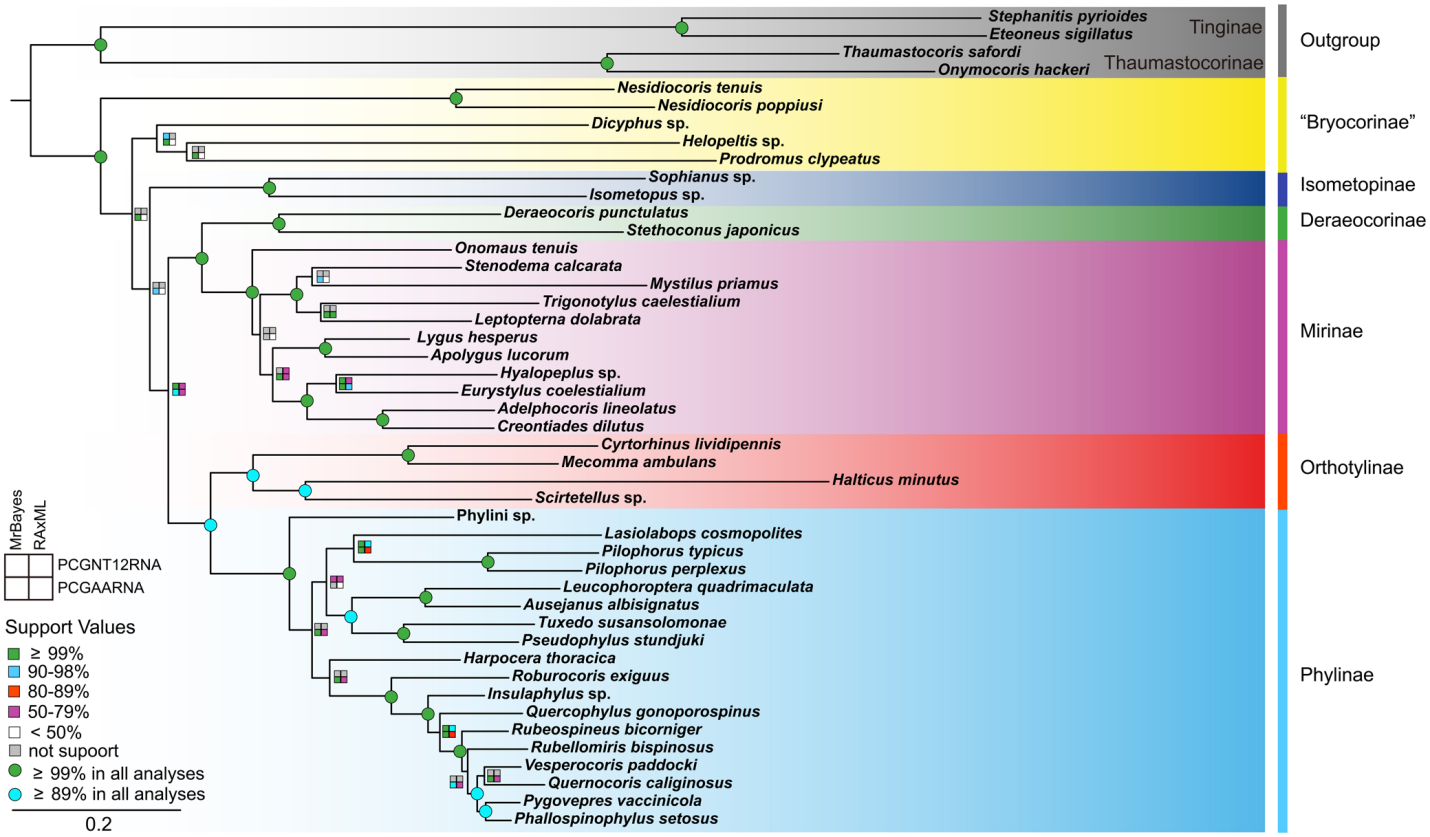
Phylogenetic tree of Miridae inferred from maximum‐likelihood analyses based on PCGAARNA datasets. Bootstrap values of maximum‐likelihood analyses and posterior probability values of Bayesian analyses are labeled around each node.

Based on the mitogenomes of 46 Miridae species representing six subfamilies, our phylogenetic analyses yielded highly consistent topologies using both Bayesian inference (BI) and maximum likelihood (ML) reconstructions (Figure 4 and Figures S5–S7). The five subfamilies, Mirinae, Orthotylinae, Deraeocorinae, Isometopinae, and Phylinae, were stably recovered as monophyletic groups across all analyses. The clade ((Deraeocorinae + Mirinae) + (Orthotylinae + Phylinae)) was consistently supported as monophyletic across all datasets and analytical approaches. In contrast, the monophyly of Bryocorinae was not supported and instead appeared as a paraphyletic group. Phylogenetic analyses of the PCGAARNA dataset recovered Isometopinae as the sister group to all other mirids except “Bryocorinae”, with a posterior probability of 96% but low bootstrap value (BS < 50%) (Figure 4). While analyses of the PCGNT12RNA dataset provided a different topology, in which Isometopinae was recovered as the sister group of all remaining mirids except *P. clypeatus* (in Bryocorinae) (Figure S5).

## 4 Discussion

This study significantly expands the available mitogenomic data for the family Miridae by sequencing and presenting the complete mitochondrial genomes of four additional genera: *Chlamydatus* sp. (Phylinae), *Deraeocoris punctulatus* (Deraeocorinae), *Scirtetellus* sp. (Orthotylinae), and *Prodromus clypeatus* (Bryocorinae). This raises the total number of taxa with a complete mitogenome in NCBI from 21 to 25, a 20% increase that provides a crucial data foundation for future studies. All mitogenomes exhibited a characteristic pattern of other heteropteran insects (Ye et al. 2022). Phylogenetic trees reconstructed from mitogenomes reveal high congruence across different analytical methods, establishing a robust foundation for advancing phylogenetic and taxonomic research. Furthermore, this study also demonstrates the high resolution of mitogenomes in elucidating relationships within the family Miridae.

### 4.1 Evolutionary Patterns and Selective Pressures

The exceptionally low evolutionary rates observed in the core oxidative phosphorylation genes (*COI*, *COII*, *COIII*, *CYTB*), as evidenced by their very low Ka/Ks ratios, confirm their utility as highly conserved phylogenetic markers for deeper taxonomic levels. This strong purifying selection explains the strong congruence and high support values for the deeper nodes in our trees, such as the clade ((Deraeocorinae + Mirinae) + (Orthotylinae + Phylinae)). Conversely, the higher evolutionary rate of genes like ATP8 may make them more informative for resolving relationships within recently radiated groups, suggesting a tiered approach for future studies targeting different taxonomic levels.

### 4.2 Bryocorinae Phylogeny

Our mitogenomic phylogenetic analysis robustly supports the paraphyly of Bryocorinae, consistent with Jung and Lee (2012) and Oh et al. (2023), and further provides the novel result that these lineages constitute the stem groups of Miridae. Specifically, Jung and Lee (2012) indicated non‐monophyly due to the separation of tribes like Dicyphini and Bryocorini in their multi‐locus phylogeny, while Oh et al. (2023) reported polyphyly results based on mitogenomic fragments and nuclear histone genes. Our study, utilizing complete mitochondrial genomes, provides enhanced phylogenetic signal and resolution, clearly recovering Bryocorinae as paraphyletic with key genera such as *Dicyphus* Fieber, 1858, placed in a sub‐basal position and forming a clade with *Prodromus* + *Helopeltis* Signoret, 1858. This topology contrasts with the isolated placement of *Prodromus* in Oh et al. (2023) and underscores the superiority of whole mitogenomic data in resolving deep nodes within rapidly radiating lineages like Miridae.

The persistent conflict between molecular datasets and morphology‐based analyses highlights the necessity of large‐scale molecular data to overcome limitations of fragmentary markers, which can obscure true evolutionary relationships. Consequently, our results reinforce the need for substantial taxonomic revision within Miridae, particularly to address the paraphyly of Bryocorinae, and argue for the adoption of phylogenomic approaches to achieve a more reasonable classification.

### 4.3 Bryocorinae Morphological Derivatives

The morphological framework proposed by Namyatova et al. (2016) suggested the monophyly of Bryocorinae based on specific character states including asymmetrical parempodia, tuberculate trichobothria, pseudopulvilli, and a short medial fracture on the hemelytron, while also establishing tribes such as Felisacini (defined by elongate head with prominent eyes, modified male genitalia with sclerotized structures, and a unique setal pattern on hemelytra) and Monaloniini (characterized by dilated tarsi with pseudopulvilli originating from the claw base membranous area, large body size, absence of a metathoracic scent gland evaporative area, and symmetrical parempodia; subsequent refinements emphasizing synapomorphies such as a supragenital bridge within the genital capsule, a posteriorly projecting metepimeron, and an impunctate mesoscutum‐scutellum sulcus).

However, the paraphyletic topology of Bryocorinae indicates that many putative morphological synapomorphies, including those defining Monaloniini, are likely homoplastic or convergent, evolving multiple times independently within Miridae rather than representing true synapomorphies for a monophyletic group. For instance, the placement of *Helopeltis*, which exhibits diagnostic Monaloniini traits like a scutellar process and asymmetrical parempodia, within a clade containing genera lacking these features underscores the convergent nature of these derivatives (Namystova and Cassis 2016). Similarly, the distribution of character states across disparate lineages in our phylogeny suggests widespread homoplasy, necessitating a fundamental re‐evaluation of which morphological characters reliably indicate common ancestry.

Consequently, our findings, aligned with those of Oh et al. (2023), present a compelling case for substantial taxonomic revision within Miridae. This is particularly true for the paraphyletic subfamilies Bryocorinae, whose current classifications are incongruent with phylogenetic evidence. To achieve a robust and stable classification, future work should integrate phylogenomic‐scale datasets with a critical re‐examination of morphological synapomorphies, emphasizing an integrated framework that combines genome‐scale molecular data with refined character assessments to resolve these long‐standing systematic issues.

### 4.4 Phylogeny of the Crown Clade

The sister relationship between Phylinae and Orthotylinae was strongly supported in this study, as evidenced by high posterior probability and bootstrap values (BS > 90%) (Figure 4). This result is consistent with those of Kim et al. (2022) and Oh et al. (2023). Notably, although Kim et al. (2022) also recovered this clade using UCE data, it received only moderate support (BS < 70%). A similar level of support was observed in the results of Oh et al. (2023). In addition, our analyses recovered Mirinae and Deraeocorinae as a well‐supported monophyletic group, a finding that aligns with the results of Oh et al. (2023). Schuh et al. (2009) ambiguously positioned Deraeocorinae near Cylapinae in combined analyses, a discrepancy potentially caused by data conflict in their sparse matrix. Crucially, although Namyatova et al. (2016) proposed this relationship based on male genitalia morphology, our analysis based on mitochondrial genome data now provides definitive corroborative evidence.

### 4.5 Research Gaps

While this study significantly increases the mitogenomic data for Miridae, taxon sampling, particularly for the critically important but missing Cylapinae subfamily and underrepresented groups like Isometopinae, remains a limitation. The unstable position of Isometopinae in our analyses, varying with the dataset, likely reflects this sparse sampling. Furthermore, the inherent limitations of a single, maternally inherited genomic marker must be considered. To build a fully resolved and robust phylogeny, future efforts should prioritize: (1) Filling the taxonomic sampling gaps in Cylapinae, while also maximizing the coverage of additional tribes in the subfamilies Bryocorinae and Isometopinae to test the extent of paraphyly and homoplasy; (2) Sequencing multi‐locus nuclear datasets to complement the mitochondrial signal and resolve conflicts between molecular and morphological data, particularly for problematic groups like Bryocorinae; (3) Re‐evaluating morphological characters through an integrated framework that combines genomic‐scale data with critical morphological reappraisal, to distinguish true synapomorphies from homoplastic traits and redefine monophyletic lineages.

## 5 Conclusion

This study represents the first phylogenetic reconstruction of Miridae based on complete mitochondrial genome data. It thus provides a robust framework and a novel reference for future studies within this hyperdiverse family. Our analyses yielded several robust and consistent findings. First, the subfamily Bryocorinae was resolved as a paraphyletic stem group of Miridae, a result that challenges its traditional taxonomic status. Second, the monophyly of the clades (Deraeocorinae + Mirinae) and (Orthotylinae + Phylinae) was strongly supported by all datasets and analytical methods. Finally, a well‐supported sister‐group relationship was identified between Mirinae and Deraeocorinae. These findings demonstrate the power of mitogenomic data, particularly when combined with critical improvements in taxon sampling. By laying a new foundation for utilizing mitogenomes to elucidate relationships within Miridae, this work provides an indispensable dataset for guiding the comprehensive taxonomic revision that the family clearly requires, especially for Bryocorinae. Future studies that incorporate broader taxon sampling along with nuclear gene data will be crucial for further clarifying the phylogeny of Miridae.

## Author Contributions

**Jia‐Dong Yin:** conceptualization (equal), investigation (lead), methodology (lead), validation (lead), writing – original draft (lead), writing – review and editing (lead). **Bo‐Lun Cai:** data curation (lead), formal analysis (lead), software (lead), validation (lead), visualization (lead), writing – original draft (supporting). **Wen‐Jun Bu:** funding acquisition (lead), supervision (lead), writing – review and editing (lead). **Qiang Xie:** conceptualization (equal), funding acquisition (lead), project administration (lead), resources (lead), supervision (lead), writing – review and editing (lead).

## Funding

This study was supported by the Science & Technology Fundamental Resources Investigation Program of China (2023FY100200) and The Natural Science Foundation of China (No. 32130014).

## Conflicts of Interest

The authors declare no conflicts of interest.

## Supporting information


**Figures S1–S7:** ece373035‐sup‐0001‐Figures.docx.

**Tables S1–S2:** ece373035‐sup‐0002‐Tables.docx.

**Data S1–S2:** ece373035‐sup‐0003‐DataS1‐S2.zip.

## Data Availability

The data that support the findings of this study are openly available as Data S1 and S2.
