# Supplementary material for: Phylogenetic Relationships of Plant Bugs Based on Mitochondrial Genomes (Heteroptera: Miridae)
Source: Ecol Evol. 2026 Feb 4;16(2):e73035. doi: 10.1002/ece3.73035 (PMC12872965; doi:10.1002/ece3.73035)
Supplement: Supplementary file 1 — Figures S1–S7: ece373035‐sup‐0001‐Figures.docx. [file ECE3-16-e73035-s001.docx]

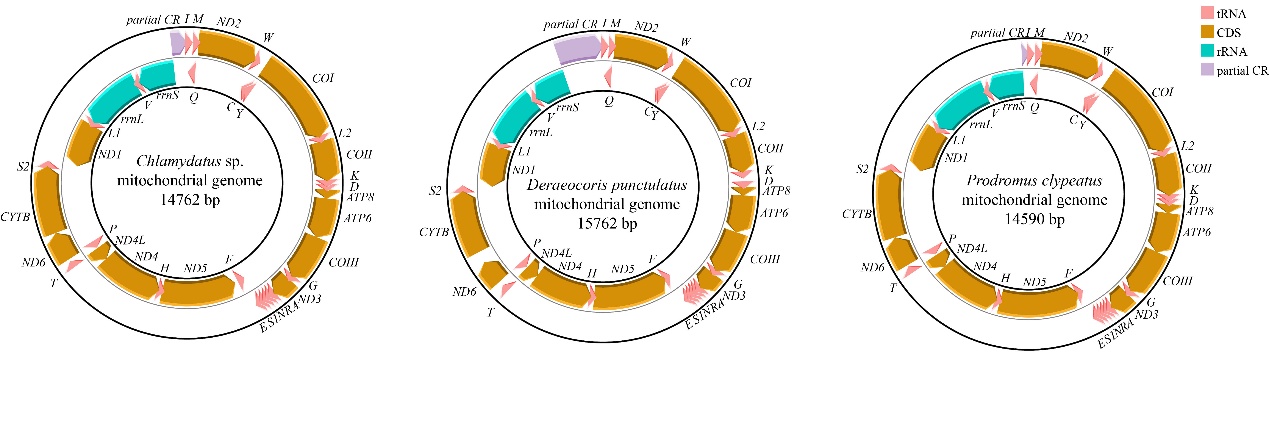


Figure S1. Circular diagram of the *Chlamydatus* sp., *Deraeocoris punctutatus* and *Prodromus clypeatus* mitogenome, including control region. The transcriptional direction is denoted by arrows.


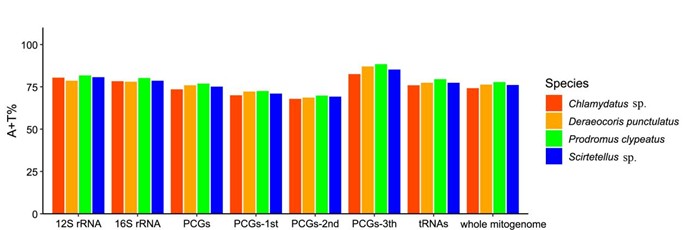


Figure S2. The A+T content of *Chlamydatus* sp., *Deraeocoris punctutatus*, *Scirtetellus* sp. and *Prodromus clypeatus* mitochondrial genomes.


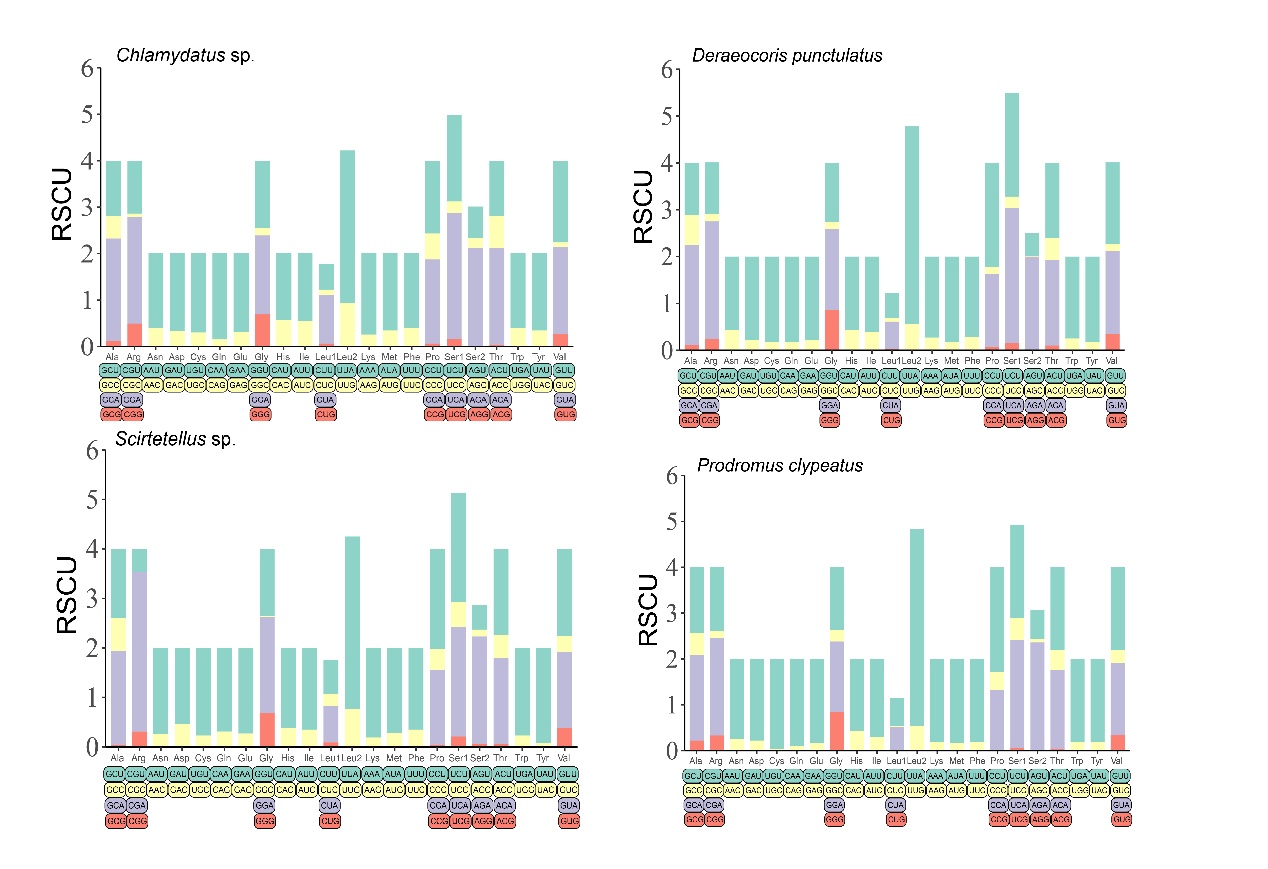


Figure S3. Relative synonymous codon usage (RSCU) of mitochondrial genomes of *Chlamydatus* sp., *Deraeocoris punctutatus*, *Scirtetellus* sp. and *Prodromus clypeatus.*


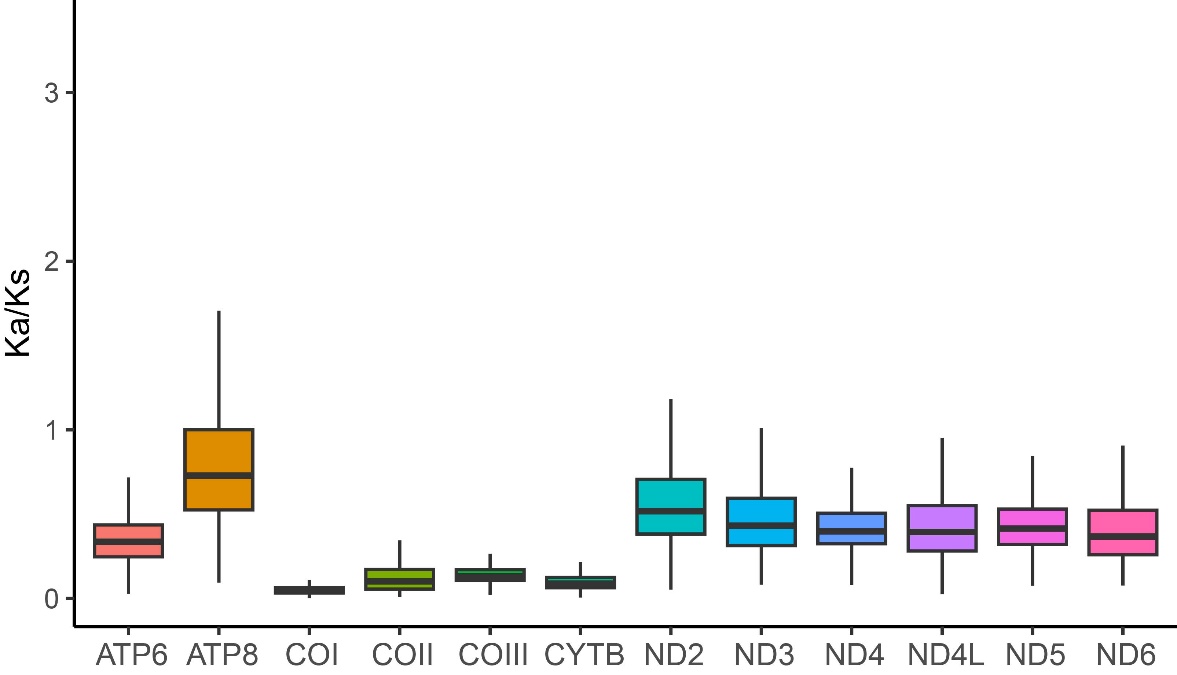


Figure S4. Average evolutionary rate of Miridae mitochondrial PCGs.


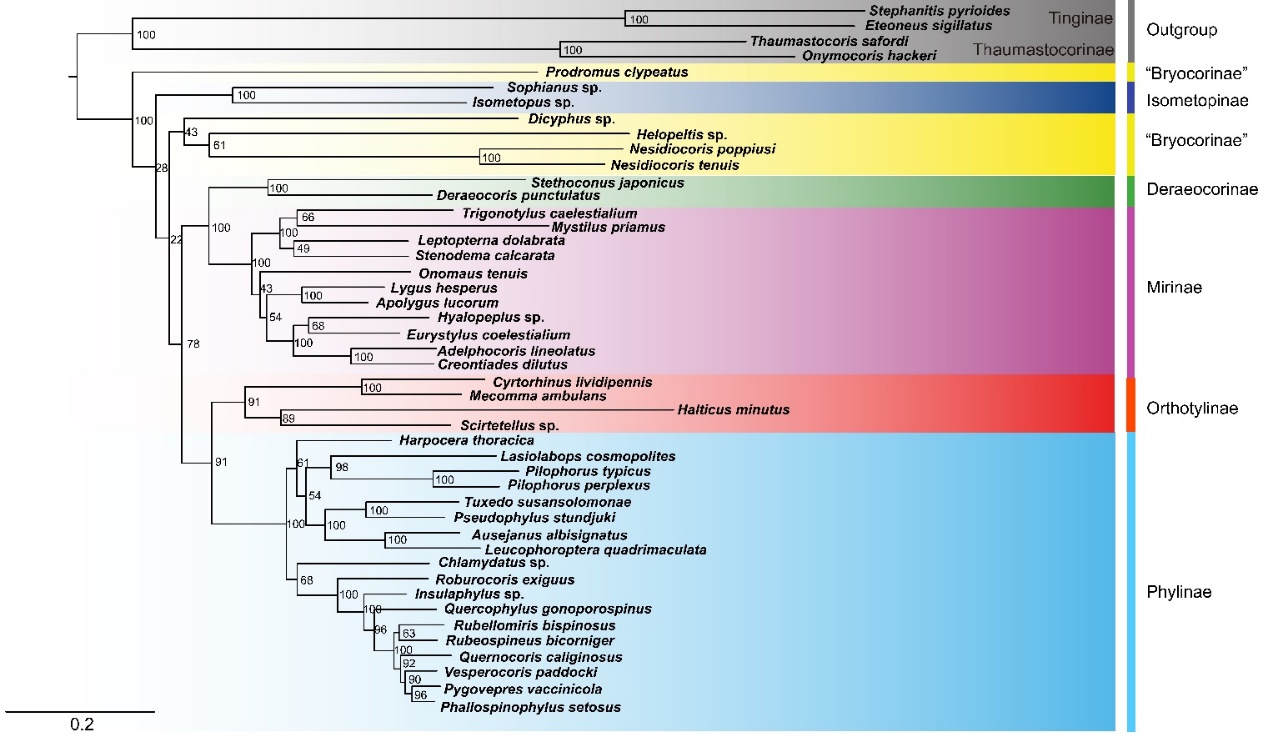


Figure S5. Phylogenetic tree inferred from PCGNT12RNA constructed using RAxML analysis. Numbers at the nodes are bootstrap values.


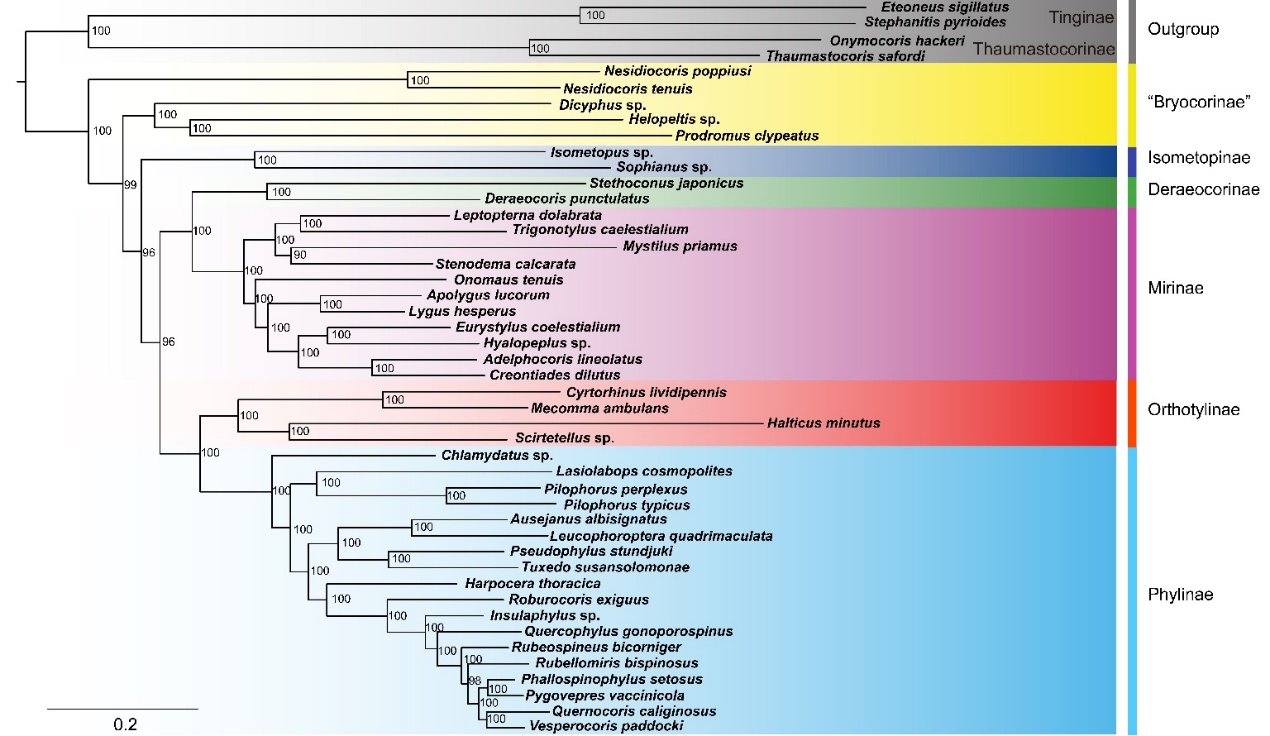


Figure S6. Phylogenetic tree inferred from PCGAARNA constructed using MrBayes analysis. Numbers at the nodes are Bayesian posterior probabilities.


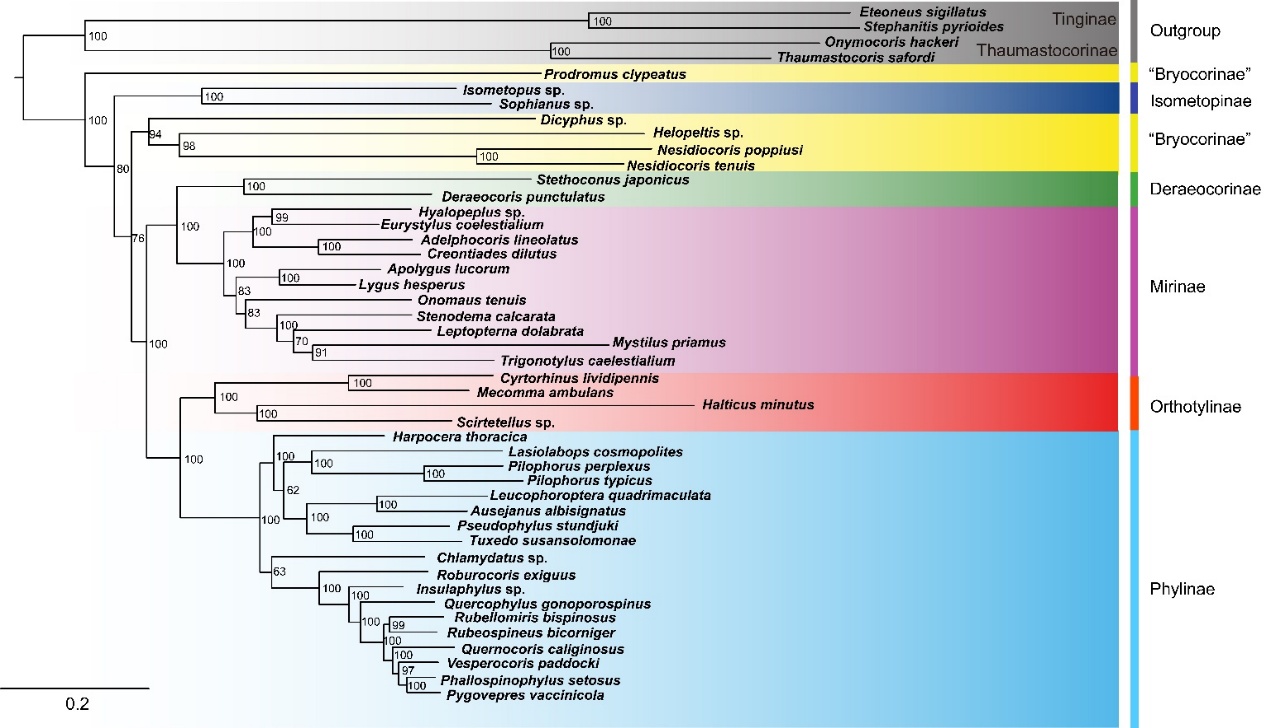


Figure S7. Phylogenetic tree inferred from PCGNT12RNA constructed using MrBayes analysis. Numbers at the nodes are Bayesian posterior probabilities.
