# Supplementary material for: Phylogenetic Relationships of Plant Bugs Based on Mitochondrial Genomes (Heteroptera: Miridae)
Source: Ecol Evol. 2026 Feb 4;16(2):e73035. doi: 10.1002/ece3.73035 (PMC12872965; doi:10.1002/ece3.73035)
Supplement: Supplementary file 2 — Tables S1–S2: ece373035‐sup‐0002‐Tables.docx. [file ECE3-16-e73035-s003.docx]

**Table S1.** The best fit partition schemes and models used for phylogenetic analyses.

| **Partitioning scheme** | **Gene** | **Model** |
| --- | --- | --- |
| RAXML-PCGAARNA-Partition 1 | ATP6_COII_COIII | mtART+F+I+G4 |
| RAXML-PCGAARNA-Partition 2 | ATP8_ND2_ND3_ND6 | mtZOA+F+I+G4 |
| RAXML-PCGAARNA-Partition 3 | COI_CYTB | mtART+F+I+G4 |
| RAXML-PCGAARNA-Partition 4 | ND1_ND4_ND4L_ND5 | mtZOA+F+I+G4 |
| RAXML-PCGAARNA-Partition 5 | 12S | GTR+F+I+G4 |
| RAXML-PCGAARNA-Partition 6 | 16S | GTR+F+I+G4 |
| RAXML-PCGAARNA-Partition 7 | 22tRNAs | GTR+F+I+G4 |
| RAXML-PCGNT12RNA-Partition 1 | ATP6 | GTR+F+I+G4 |
| RAXML-PCGNT12RNA-Partition 2 | ATP8 | GTR+F+I+G4 |
| RAXML-PCGNT12RNA-Partition 4 | COI | GTR+F+I+G4 |
| RAXML-PCGNT12RNA-Partition 5 | COII | GTR+F+I+G4 |
| RAXML-PCGNT12RNA-Partition 6 | COIII_CYTB | GTR+F+I+G4 |
| RAXML-PCGNT12RNA-Partition 7 | ND1 | GTR+F+I+G4 |
| RAXML-PCGNT12RNA-Partition 8 | ND2_ND6 | GTR+F+I+G4 |
| RAXML-PCGNT12RNA-Partition 9 | ND3 | GTR+F+I+G4 |
| RAXML-PCGNT12RNA-Partition 10 | ND4_ND5 | GTR+F+I+G4 |
| RAXML-PCGNT12RNA-Partition 11 | ND4L | GTR+F+I+G4 |
| RAXML-PCGNT12RNA-Partition 12 | 12S | GTR+F+I+G4 |
| RAXML-PCGNT12RNA-Partition 13 | 16S | GTR+F+I+G4 |
| RAXML-PCGNT12RNA-Partition 14 | 22tRNAs | GTR+F+I+G4 |
| MrBayes-PCGAARNA-Partition 1 | ATP6_COII_COIII | mtART+F+R5 |
| MrBayes-PCGAARNA-Partition 2 | ATP8_ND2_ND3_ND6 | mtMet+F+R5 |
| MrBayes-PCGAARNA-Partition 3 | COI_CYTB | mtART+F+R5 |
| MrBayes-PCGAARNA-Partition 4 | ND1_ND4_ND4L_ND5 | mtZOA+F+R6 |
| MrBayes-PCGAARNA-Partition 5 | 12S | GTR+F+R4 |
| MrBayes-PCGAARNA-Partition 6 | 16S | GTR+F+I+G4 |
| MrBayes-PCGAARNA-Partition 7 | 22tRNAs | GTR+F+R5 |
| MrBayes-PCGNT12RNA-Partition 1 | ATP6_ND3 | GTR+F+R5 |
| MrBayes-PCGNT12RNA-Partition 2 | ATP8 | TPM2+F+I+G4 |
| MrBayes-PCGNT12RNA-Partition 4 | COI | GTR+F+I+G4 |
| MrBayes-PCGNT12RNA-Partition 5 | COII | GTR+F+I+G4 |
| MrBayes-PCGNT12RNA-Partition 6 | COIII_CYTB | GTR+F+R4 |
| MrBayes-PCGNT12RNA-Partition 7 | ND1 | K3Pu+F+R5 |
| MrBayes-PCGNT12RNA-Partition 8 | ND2_ND6 | GTR+F+R4 |
| MrBayes-PCGNT12RNA-Partition 10 | ND4_ND5 | GTR+F+R5 |
| MrBayes-PCGNT12RNA-Partition 11 | ND4L | K3Pu+F+R3 |
| MrBayes-PCGNT12RNA-Partition 12 | 12S | GTR+F+R4 |
| MrBayes-PCGNT12RNA-Partition 13 | 16S | GTR+F+R5 |
| MrBayes-PCGNT12RNA-Partition 14 | 22tRNAs | GTR+F+R5 |

**Table S2.** Mitochondrial genome statistics for the *Chlamydatus* sp., *Deraeocoris punctutatus*, *Scirtetellus* sp. and *Prodromus clypeatus*. AT-skew and GC-skew were measured for the 37 genes except the control regions.

| Species | whole mitogenome（A+T %） | 12S rRNA | 16S rRNA | tRNAs | PCGs | PCGs-1st | PCGs-2nd | PCGs-3th | AT-skew | GC-skew |
| --- | --- | --- | --- | --- | --- | --- | --- | --- | --- | --- |
| *Chlamydatus* sp. | 74.2 | 80.4 | 78.3 | 75.9 | 73.5 | 70 | 67.9 | 82.5 | 0.16 | -0.19 |
| *Deraeocoris punctulatus* | 76.3 | 78.6 | 78 | 77.4 | 75.9 | 72.2 | 68.6 | 87 | 0.14 | -0.16 |
| *Scirtetellus* sp. | 76.1 | 80.7 | 78.6 | 77.4 | 75.1 | 71 | 69.2 | 85.2 | 0.11 | -0.17 |
| *Prodromus clypeatus* | 77.8 | 81.7 | 80.2 | 79.5 | 76.9 | 72.5 | 69.8 | 88.4 | 0.09 | -0.16 |
